# Artificial Intelligence in the Diagnosis of Cholesteatoma: A Systematic Review of Current Evidence

**DOI:** 10.7759/cureus.96154

**Published:** 2025-11-05

**Authors:** Shaqra M Shabi, Lama B Almutairi, Abdulkarim N Moafa, Rahaf A Omer, Ahmed M Alghamdi, Hussam S Alhemaidi, Hanouf H Alhamyani, Aws A Abualiat, Atallah A Alamdi, Marwan S Alkhaibari, Layla H Al Khairat

**Affiliations:** 1 Department of Surgery, Jazan University Hospital, Jazan, SAU; 2 College of Medicine, Qassim University, Qassim, SAU; 3 College of Medicine, Jazan University, Jazan, SAU; 4 College of Medicine, Al Baha University, Al Baha, SAU; 5 College of Medicine, Taibah University, Al Madinah, SAU; 6 College of Medicine, Taif University, Taif, SAU; 7 College of Medicine and Surgery, University of Bisha, Bisha, SAU; 8 Faculty of Medicine, University of Tabuk, Tabuk, SAU

**Keywords:** artificial intelligence, cholesteatoma, computed tomography, convolutional neural network, diagnostic imaging, machine learning, magnetic resonance imaging, otology, otoscopy, systematic review

## Abstract

This systematic review evaluated the application of artificial intelligence (AI) in the imaging-based diagnosis of cholesteatoma. A comprehensive search of PubMed, Scopus, and Web of Science up to September 2025 identified 8,160 records, of which 7 studies met the inclusion criteria. Five studies used temporal bone CT, one investigated otoscopic images, and one assessed CT-based staging of mastoid extension. Reported internal validation accuracies were generally high (>90%), particularly with convolutional neural network (CNN) architectures, while external validations showed moderate performance (78-88%). A multicenter 3D convolutional neural network (CNN) with automated region-of-interest detection demonstrated consistent generalizability and aided surgical planning in prospective testing. The otoscopy-based model achieved high accuracy for differentiating cholesteatoma from normal membranes (>98%), though performance decreased when distinguishing it from other middle ear pathologies. Across studies, AI models often performed comparably to human readers, and explainability tools highlighted relevant diagnostic features. Most studies were retrospective and single-center, with limited external validation. Overall, AI shows strong potential for cholesteatoma diagnosis using CT and otoscopic imaging, but multicenter prospective studies with standardized evaluation and clinical impact assessment are needed before routine implementation.

## Introduction and background

Cholesteatoma is a destructive lesion of the middle ear and mastoid characterized by keratinizing squamous epithelium, which can cause bone erosion, hearing loss, vestibular dysfunction, and potentially life-threatening intracranial complications if untreated [1,2]. Although histologically benign, its invasive behavior necessitates timely and accurate diagnosis to guide surgical management. Differentiating cholesteatoma from other chronic otitis media (COM) conditions is essential, as treatment strategies differ substantially [3,4].

High-resolution computed tomography (HRCT) is routinely used to assess temporal bone anatomy and detect bony erosion, but has limited ability to distinguish soft tissue pathologies, such as cholesteatoma, from other inflammatory lesions. Diffusion-weighted magnetic resonance imaging (DWI-MRI), particularly non-echo planar imaging, offers higher specificity for cholesteatoma detection but is constrained by cost, limited availability, and artifact sensitivity [3,4]. Consequently, preoperative diagnosis remains challenging, and misclassification may result in unnecessary or delayed surgeries.

Artificial intelligence (AI), particularly deep learning (DL) techniques, has shown promise in medical imaging by automatically extracting complex features and improving diagnostic accuracy [1,3,5]. In otology, AI has been applied to CT, MRI, and endoscopic imaging for conditions such as otosclerosis, otitis media, and cholesteatoma. Leveraging convolutional neural networks (CNNs) and other machine learning (ML) algorithms, AI can augment radiologic and surgical decision-making, especially in settings where imaging expertise is limited [4,6].

Despite growing interest, studies on AI for cholesteatoma diagnosis vary widely in methodology, imaging modalities, model architectures, and validation strategies. While some report high diagnostic accuracies (>90%) on internal datasets, most lack external validation or prospective assessment, raising concerns about generalizability. Additionally, explainability, clinical integration, and comparisons with human experts are inconsistently addressed [2,3].

This systematic review aims to critically evaluate and synthesize emerging evidence on AI applications in cholesteatoma diagnosis. Specifically, it assesses study designs, populations, imaging modalities, model architectures, validation strategies, diagnostic performance, and reported clinical utility. By consolidating these findings, the review provides an evidence-based overview of AI’s current role in cholesteatoma diagnosis and identifies priorities for future research to support clinical translation.

## Review

Methodology

Literature Search Strategy

**Figure 1:**
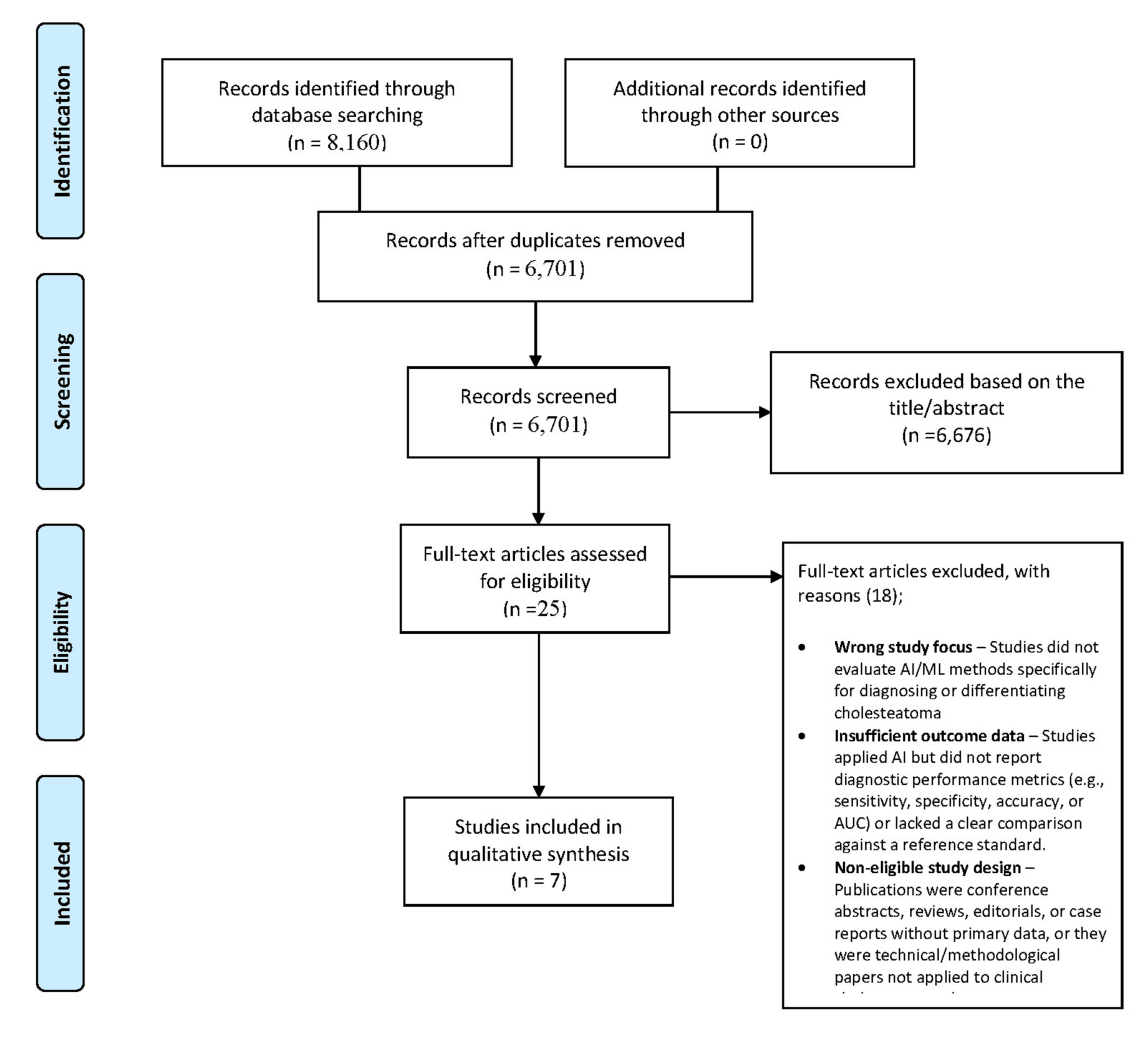
PRISMA flow diagram of the study selection process PRISMA: Preferred Reporting Items for Systematic reviews and Meta-Analyses; AUC: area under the curve

We followed Preferred Reporting Items for Systematic reviews and Meta-Analyses (PRISMA) guidance [7] and searched PubMed, Web of Science, and Scopus from inception to 10 September 2025 using controlled vocabulary and free-text terms (e.g., “cholesteatoma,” “artificial intelligence,” “machine learning,” “deep learning,” “neural network,” “CNN,” “support vector machine,” “computer-aided diagnosis”), adapted to each database’s syntax and combined with Boolean operators. Searches were limited to English and human studies. Reference lists of included papers were hand-searched for additional records (Figure 1).

Eligibility Criteria

Using the PICOS (Population, Intervention, Comparison, Outcomes, and Study) approach [8], we included studies that: (1) enrolled patients with suspected or confirmed cholesteatoma; (2) applied AI/ML/DL models to CT, MRI/DWI, or otoscopic images for diagnosis; (3) compared AI outputs against a reference standard (surgical/histopathology, expert readers, or conventional imaging); and (4) reported diagnostic performance (sensitivity, specificity, accuracy, area under the curve (AUC) or equivalent). Retrospective and prospective diagnostic accuracy studies were eligible. We excluded studies not focused on cholesteatoma diagnosis, those lacking diagnostic performance data, conference abstracts, reviews, case reports, editorials, and non-English publications.

Study Selection and Data Extraction

Two reviewers independently screened titles/abstracts and then full texts against the eligibility criteria. Disagreements were resolved by discussion with a third reviewer. Two reviewers independently extracted data into a predesigned table capturing study setting and design, population, reference standard, imaging modality, AI model and architecture, evaluation/validation strategy, diagnostic metrics, explainability, and reported clinical applicability. Discrepancies were adjudicated by a third reviewer.

Quality Appraisal

Two reviewers independently assessed risk of bias using QUADAS-2 [9], supplemented with AI-specific considerations from PROBAST-AI (data leakage, validation strategy, transparency) [10]. Domains were rated low/high/unclear risk; disputes were resolved by consensus with a third reviewer.

Results

Study Selection

Database searches identified 8,160 records (PubMed: 4,477; Scopus: 2,394; Web of Science: 1,289). After removing duplicates, 6,701 unique records remained. Title and abstract screening excluded 6,676 irrelevant studies, leaving 25 full texts for review. Eighteen were excluded for reasons such as unrelated focus, lack of diagnostic performance data, or non-original format. Seven studies were included in the qualitative synthesis (Table 1) [1-6,11].

**Table 1:** Artificial intelligence models for the diagnosis and staging of cholesteatoma This table summarizes the key characteristics, imaging modalities, AI model types, evaluation methods, diagnostic performance metrics, explainability techniques, and clinical use cases of studies investigating artificial intelligence (AI) for the diagnosis or preoperative assessment of cholesteatoma. Data include study setting, design, population, cases and controls, reference standards, imaging modalities, AI architectures, training/validation strategies, external validation, comparator methods, sensitivity, specificity, accuracy, area under the receiver operating characteristic curve (AUROC), additional performance metrics (precision, recall, F1 score, positive predictive value, negative predictive value), explainability approaches (e.g., Grad-CAM, heatmaps), and proposed clinical applications. CHO, cholesteatoma; COM, chronic otitis media; CSOM, chronic suppurative otitis media; CT, computed tomography; DWI, diffusion-weighted imaging; HRCT, high-resolution CT; CNN, convolutional neural network; MLP, multilayer perceptron; SVM, support vector machine; k-NN, k-nearest neighbors; AUROC, area under the receiver operating characteristic curve; Sn, sensitivity; Sp, specificity; Acc, accuracy; PPV, positive predictive value; NPV, negative predictive value; ROI, region of interest; XAI, explainable artificial intelligence; Grad-CAM, gradient-weighted class activation mapping

Study	Country / Setting	Study Design	Population (n, Age Group, Sex)	Cases / Controls	Reference Standard	Imaging Modality	AI Model Type / Architecture	Model Evaluation & Outcomes (Training/Validation, External Validation, Comparator, Sensitivity, Specificity, Accuracy, AUROC, Other Metrics, Explainability, Clinical Use Case)
Eroğlu [1]	Turkey – Fırat University	Retrospective cohort	n=75 (adults; sex not specified)	41 CHO, 34 chronic otitis without CHO; 45 normal controls for AI dataset	Surgical confirmation (intraoperative findings)	CT (temporal bone, multidetector, axial & coronal, bone window) and MRI (DWI and other sequences)	Pretrained CNNs: AlexNet, GoogLeNet, MobileNetV2, ResNet50, DarkNet53, DenseNet201	Training/Validation: 80% training, 20% testing. External validation: None. Comparator: MRI (DWI). Performance: DenseNet201, highest accuracy: 90.99%; MobileNetV2, lowest: 83.3%; MRI, accuracy: 88%, sensitivity: 87.8%, specificity: 88.23%. Other metrics: DenseNet201, sensitivity: 90–92%, specificity: 95–96%, F1 ≈91%. Explainability: Not reported. Clinical use case: Aid CT-based cholesteatoma detection vs MRI.
Takahashi [2]	Japan – Jikei University School of Medicine, Tokyo	Retrospective, single-center study	n=164 (13–82 yrs; mean 42.0 ±15.3; 104M, 60F)	80 M+, 84 M–	Intraoperative + histopathology	HRCT (Siemens SOMATOM, 64-MDCT, 0.6 mm slice)	MobileNetV2 (88 layers, 3.5M parameters)	Training/Validation: 8-fold CV, 24 random augmentations, ensemble of 192 models. External validation: None. Comparator: 15 otolaryngologists. Performance: Ensemble best – Acc 81.14%, Sn 84.95%, Sp 77.33%, AUC 0.837; Single-image ~75%. Human benchmark: Avg 73.41% Acc, Sn 83.17%, Sp 64.13%. Explainability: Cropping-based analysis. Clinical use case: Preoperative mastoid extension prediction to guide surgical approach.
Ayral [3]	Turkey – Dicle University Faculty of Medicine	Retrospective, single-center diagnostic study	n=300 (18–65 years; sex not specified)	100 CHO, 100 non-CHO COM, 100 controls	Histopathology (CHO); imaging confirmation for controls	CT (temporal bone, axial & coronal, bone window)	Deep learning CNNs: ResNet50, MobileNetV2 (transfer learning)	Training/Validation: 80% training, 15% validation, 20% test; Shuffle algorithm. External validation: None. Comparator: None (AI models compared with each other). Performance: ResNet50 – Accuracy 93.3% (CHO 100%, N-CHO 90%, control 90%); MobileNetV2 – Accuracy 86.7% (CHO 95%, N-CHO 85%, control 80%). Other Metrics: Precision/Recall/F1 – ResNet50 (CHO P=1.00, R=0.95, F1=0.98); MobileNetV2 (CHO P=0.95, R=0.90, F1=0.93). Explainability: Grad-CAM applied. Clinical use case: Assist in the differential diagnosis of COM with vs. without cholesteatoma, reduce radiologist workload, aid treatment planning.
Tseng [4]	USA – Rutgers New Jersey Medical School	Retrospective image dataset study	n=834 otoscopic images: 197 CHO, 457 abnormal non-CHO, 180 normal (demographics not reported)	197 CHO vs 637 non-CHO	Surgical confirmation for CHO; expert labeling for others	Otoscopy (Karl Storz Tele-Otoscope, 1920×1080)	8 pretrained CNNs (VGG19, MobileNetV2, DenseNet201, InceptionV3, ResNet152V2, Xception, InceptionResNetV2, NASNetLarge); transfer learning	Training/Validation: 80% training, 10% validation, 10% testing, 100 epochs, dropout 0.2, Adam. External validation: None. Comparator: AI models compared with each other. Performance – CHO vs Normal: DenseNet201 Acc 98.5%, Sn 100%, Sp 97.1%, AUROC 0.9991; CHO vs Abnormal non-CHO: DenseNet201 Acc 90.1%, Sn 88.9%, Sp 90.5%, AUROC 0.928; CHO vs Non-CHO: Xception Acc 90.4%, Sn 63.6%, Sp 96.5%, AUROC 0.936; NASNetLarge AUROC 0.961. Other Metrics: PPV/NPV varied. Explainability: Intermediate activations heatmaps. Clinical use case: Automated otoscopic CHO diagnosis; screening/triage, teaching tool.
Chen [5]	China (Eye & ENT Hospital, Fudan University; Union Hospital, Wuhan) + USA (Vanderbilt Univ.)	Retrospective multicenter model development with prospective validation	Internal: 1661 patients, 3153 ears (mean 41.1 ±16.6, 50.1% male); External: 108 patients, 211 ears (mean 39.8 ±14.0, 45.4% male); Prospective: 121 patients (mean 46.8 ±16.1, 40.5% male)	Internal: 728 CHO, 1011 CSOM, 1130 normal, 284 other COM; External: 30 CHO, 69 CSOM, 101 normal, 11 other	Operative + pathology for operated ears; clinical + imaging + audiometry consensus for unoperated ears	Temporal bone CT (Siemens SOMATOM, 0.6–0.75 mm slice)	Automated 3D CNN (YOLOv5 ROI extraction + 3D CNN classifier; 4 conv, 2 dense blocks, dropout, softmax); Grad-CAM for explainability	Training/Validation: 5-fold CV, Adam optimizer, early stopping. External validation: Wuhan dataset. Comparator: 2D CNN (Inception-V3) + 7 human experts. Performance (Task 1, COM vs normal): Internal – AUC 0.96, Acc 87.8%, Sn 85.3%, Sp 91.3%; External – AUC 0.93, Acc 84.3%, Sn 75.6%, Sp 93.4%. Task 2 (CHO vs non-CHO): Internal – AUC 0.85, Acc 78.3%, Sn 80.8%, Sp 77.0%; External – AUC 0.83, Acc 81.3%, Sn 61.4%, Sp 87.8%. Prospective cohort: Accuracy 81.8%, aided surgical planning in 90.1% of cases. Explainability: Grad-CAM 3D heatmaps. Clinical use case: Automated, explainable CT-based COM/CHO diagnosis; reduce MRI need; support surgery.
Ouattassi [6]	Morocco – Hassan II University Hospital, Fez	Retrospective, observational case-control	n=212 (102 CHO, 110 SCOM; sex not specified)	102 CHO vs 110 SCOM; 3,398 CT images (561 CHO, 2,837 SCOM)	Surgical confirmation (operative findings)	Temporal bone CT (axial, 1 mm slice, 32-channel BrightSpeed scanner)	Feature extraction with Inception-V3 CNN → supervised ML models: kNN, Neural Network (MLP), Logistic Regression, SVM, Random Forest	Training/Validation: 70% training, 20% testing, 10% validation; 10-fold CV, grid search; oversampling for class imbalance. External validation: Independent dataset of 125 cases. Performance: Internal CV – Neural Network & kNN best (AUC 1.000, Acc 99.6–99.7%, F1 0.996–0.997); Logistic Regression AUC 0.998, Acc 98.3%; SVM AUC 0.997, Acc 97.5%; Random Forest Acc 92.0%. External validation: Neural Network 4 errors, Logistic Regression 5, SVM 7, Random Forest 28, kNN 45. Explainability: Logistic Regression interpretable via L1 regularization; no XAI for other models. Clinical use case: Differentiate cholesteatoma from suppurative COM on CT, improve specificity.
Ouattassi [11]	Morocco – Hassan II University Hospital, Fez	Retrospective case–control	n=237 (122 CHO, 115 CSOM; sex not specified)	122 CHO, 115 CSOM; 5,390 CT images	Surgical confirmation	Temporal bone HRCT (1 mm slice, 32-channel GE BrightSpeed)	Inception V3 CNN → ML classifiers: k-NN, SVM, Random Forest, Neural Network (MLP, ReLU, Adam)	Training/Validation: 80% training, 20% validation; 5-fold CV. External validation: 125 independent images. Performance (CV): k-NN Acc 98.7%, F1 0.987, AUROC 0.999; Neural Network Acc 97.4%, F1 0.974, AUROC 0.999; SVM Acc 89.6%, F1 0.897, AUROC 0.987; Random Forest Acc 66.5%, F1 0.661, AUROC 0.865. External validation: Neural Network and SVM misclassified 3 and 2/125 images. Explainability: Not reported. Clinical use case: Differentiate CHO vs CSOM on CT, compare AI models internally.

Study Characteristics

The included studies varied in design, population, and imaging modality. Most were retrospective, single-center, case-control or cohort studies [1,3,11], while one included multicenter prospective validation [5]. Sample sizes ranged from under 100 to over 3,000 ears [5]. Six studies analyzed temporal bone CT images [1-3,5,6,11], and one evaluated otoscopic images [10]. CT-based studies typically used surgical or histopathological confirmation as the reference standard, while the otoscopy study combined surgical confirmation for cholesteatoma with expert labeling for other categories [4].

AI approaches ranged from deep learning CNNs, such as ResNet, DenseNet, and MobileNet [1,5], to hybrid models using feature extraction and classical machine learning algorithms, including SVM, Random Forest, k-NN, and neural networks [5,11]. Most employed internal validation via cross-validation or train-test splits [1-3,6]; a few included external validation [5,11] or prospective testing [5]. Explainability tools, such as Grad-CAM, were inconsistently applied but, when used, highlighted relevant anatomical features [5]. Reported applications included differentiating COM with and without cholesteatoma, staging disease extent, assisting surgical planning, and triaging patients in non-specialist settings.

Quality Assessment

Overall study quality was variable (Table 2). Most were at high risk of bias in patient selection due to retrospective, single-center designs [1,3,4,6,11]. Chen et al. and Takahashi et al. demonstrated stronger designs, with consecutive sampling and multicenter or prospective validation [2,5]. Reference standards were generally robust, relying on surgical or histopathological confirmation, and flow and timing were low risk across all studies.

**Table 2:** Risk of bias assessment of artificial intelligence studies for cholesteatoma diagnosis This table summarizes the methodological quality and potential sources of bias in studies applying artificial intelligence (AI) for cholesteatoma diagnosis, assessed using the QUADAS-2 framework with AI-specific adaptations. Domains include patient selection, index test (AI model), reference standard, flow and timing, and AI-specific risks such as data leakage, validation strategy, and transparency. The overall risk of bias reflects the cumulative assessment across these domains, guiding interpretation of study reliability and generalizability. AI, artificial intelligence; CHO, cholesteatoma; COM, chronic otitis media; CSOM, chronic suppurative otitis media; CT, computed tomography; CNN, convolutional neural network; MLP, multilayer perceptron; SVM, support vector machine; k-NN, k-nearest neighbors; HRCT, high-resolution CT; ROI, region of interest; XAI, explainable artificial intelligence; Grad-CAM, gradient-weighted class activation mapping; Sn, sensitivity; Sp, specificity; Acc, accuracy; AUC, area under the receiver operating characteristic curve.

Study	Patient Selection	Index Test (AI Model)	Reference Standard	Flow & Timing	AI-Specific Risks (Data Leakage, Validation, Transparency)	Overall Risk of Bias
Eroğlu et al. [1]	High risk	Unclear risk	Low risk	Low risk	High risk	High
Takahashi et al. [2]	Low risk	Low risk	Low risk	Low risk	High risk	Moderate
Ayral et al. [3]	High risk	Unclear risk	Low risk	Low risk	High risk	High
Tseng et al. [4]	High risk	Low risk	Low risk	Low risk	High risk	High
Chen et al. [5]	Low risk	Low risk	Low risk	Low risk	Low risk	Low
Ouattassi et al. [6]	High risk	Low risk	Low risk	Low risk	Unclear risk	High
Ouattassi et al. [11]	High risk	Low risk	Low risk	Low risk	Unclear risk	High

AI-specific risks are primarily related to limited external validation and potential overfitting [1,3,4]. Ouattassi et al. attempted external validation but observed reduced model performance, underscoring generalizability issues [11]. Chen et al. had the lowest overall bias due to multicenter design, 3D modeling, and prospective evaluation [5]. The study by Takahashi et al. was considered a moderate risk overall due to a lack of external validation [2].

CT-Based AI for Differentiating Cholesteatoma

Several studies applied AI to temporal bone CT to distinguish cholesteatoma (CHO) from COM or normal ears. Ayral et al. trained ResNet50 and MobileNetV2 models on 300 CT scans, achieving accuracies of 93.3% and 86.7%, respectively [3]. Eroğlu et al. found DenseNet201 reached 90.99% accuracy, comparable to diffusion-weighted MRI (DWI) (88% accuracy, 87.8% sensitivity, 88.2% specificity) [1]. Ouattassi et al. used Inception-V3 embeddings with classical ML classifiers, achieving up to 98.7% accuracy and area under the receiver operating characteristic curve (AUROC) values above 0.98, while random forest performed worse (66.5% accuracy) [11]. In a follow-up study, Ouattassi et al. validated models on an independent dataset of 125 images, with neural networks and logistic regression maintaining high performance, though k-NN showed reduced generalization [6]. Chen et al. developed a 3D CNN with automated region-of-interest detection using multicenter data, achieving 87.8% internal and 84.3% external accuracy (AUROC 0.93-0.96) [5]. Prospective testing confirmed clinical utility, aiding surgical planning in 90.1% of cases.

CT-Based AI for Disease Staging

Takahashi et al. used MobileNetV2 ensembles to predict mastoid extension in pars flaccida cholesteatoma, achieving 81.1% accuracy (sensitivity 84.9%, specificity 77.3%, AUROC 0.837) [2]. The model outperformed otolaryngologists (73.4% accuracy), indicating potential utility in preoperative staging and surgical planning.

Otoscopy-Based AI for Detecting Cholesteatoma

Tseng et al. evaluated CNNs using otoscopic images [4]. DenseNet201 achieved 98.5% accuracy (AUROC 0.999) for differentiating CHO from normal tympanic membranes but declined to 90.1% (AUROC 0.928) for CHO versus abnormal non-CHO ears. For the combined classification of CHO versus all non-CHO ears, Xception achieved 90.4% accuracy with an AUROC of up to 0.961, suggesting strong potential for screening or triage applications, though reduced specificity for complex differentials.

Cross-Study Patterns

Single-center studies commonly reported high internal accuracies (>90%), whereas externally validated models achieved more conservative but clinically credible performance (78-88%) [5,6,11]. Models incorporating 3D context and automated region-of-interest extraction generalized better than 2D CNNs or non-parametric approaches prone to overfitting. Explainability methods consistently localized relevant anatomical regions, supporting interpretability and clinical trust. Across studies, AI performance equaled or exceeded that of human experts in diagnostic and staging tasks [2,5].

## Conclusions

Artificial intelligence shows considerable potential to enhance the diagnosis and management of cholesteatoma, offering tools that can support preoperative staging, surgical planning, and triage in clinical and primary care settings. Models that integrate robust validation, three-dimensional analysis, and automated region-of-interest detection demonstrate more consistent and generalizable performance. However, current evidence is limited by retrospective designs, single-center cohorts, and variable reporting of model interpretability and calibration. Future work should focus on prospective, multicenter studies with standardized outcomes and evaluation of clinical impact to ensure AI is safely and effectively integrated alongside clinician expertise.
